# Rapid adsorptive removal of eosin yellow and methyl orange using zeolite Y

**DOI:** 10.1038/s41598-023-48675-4

**Published:** 2023-12-04

**Authors:** John Busayo Adeoye, David Ololade Balogun, Oghenefejiro Jeshurun Etemire, Princewill Nnaneme Ezeh, Yie Hua Tan, Nabisab Mujawar Mubarak

**Affiliations:** 1 Department of Chemical and Energy Engineering, Faculty of Engineering and Science, Curtin University Malaysia, CDT 250, 98009 Miri, Sarawak Malaysia; 2 Chemical Engineering Department, Faculty of Engineering, Landmark University, P.M.B 1001, Omu-Aran, Kwara Nigeria; 3 Petroleum and Chemical Engineering, Faculty of Engineering, Universiti Teknologi Brunei, Bandar Seri Begawan, BE1410 Brunei Darussalam; 4 Department of Biosciences, Saveetha School of Engineering, Saveetha Institute of Medical and Technical Sciences, Chennai, India

**Keywords:** Environmental sciences, Environmental social sciences, Energy science and technology, Materials science

## Abstract

In this study, zeolite Y was synthesised using a novel method. The heat generated from the reaction of H_2_SO_4_ with metakaolin was used as a heat source instead of applying external heat for the dealuminated process. The synthesised zeolite Y produced was analysed by scanning electron microscope (SEM), X-ray diffraction (XRD), Fourier-infrared spectroscopy (FTIR), energy dispersive X-ray spectroscopy (EDS) and Brunauer–Emmett–Teller (BET). Zeolite Y synthesis was mesoporous because of its pore diameter (30.53 nm), as shown in the BET results. Surface area and pore size decrease after adsorption due to dye deposition on the adsorbent’s surface. FTIR has bonds like O–H, C–H, –CH_3_, and –COOH responsible for adsorption. The maximum adsorption capacity of eosin yellow (EY) and methyl orange (MO) on to zeolite Y by the Langmuir isotherm was 52.91 mg/g and 20.62 mg/g respectively, at pH 2.5 and 8 for EY and MO dye. The batch adsorption studies were conducted, and the influence of different parameters (i.e., adsorbent dose, adsorption time, initial dye concentration, pH and temperature) was investigated. Experimental data were analysed by two linear model equations (Langmuir and Freundlich isotherms), and it was found that the Langmuir isotherm model best describes the adsorption data for methyl orange and Freundlich isotherm for eosin yellow, respectively. Adsorption rate constants were determined using linear pseudo-first-order and pseudo-second-order. The results showed that MO and EY dye adsorption onto zeolite Y followed a pseudo-second-order kinetic model. Thermodynamic studies show that adsorption was an exothermic reaction (enthalpy < 0) and feasible ($(Gibbs free energy)<0$) at various temperatures under investigation.

## Introduction

Synthetic dyes are used in the textile, paper, plastic, rubber, food, pharmaceutical, and cosmetic industries, and the discharge of dye wastewater to the environment and ecosystem at large is hazardous^1, 2^. Dyes are visible and undesirable at very low concentrations, and they significantly impact aquatic life due to reduced light penetration^3^. According to^4^, some dyes are hazardous and even carcinogenic. Each year, roughly 12% of synthetic dyes are lost during manufacturing and processing, and 20% reach the environment via effluents created by residual industrial wastewater treatment^5^. The dye industry is a significant industrial source of environmental pollution since it uses large amounts of water and, as a result, discharges effluent comprising a range of synthetic pigments. This accounts for up to 20% of all worldwide generated effluent^6–10^. Due to their high toxicity, strong colour and degradation resistance, dyes in wastewater have attracted much attention in recent years as their wastewater has increased, causing more significant damage to water resources^11–13^. This industrial release might negatively impact the ecosystem wildlife and cause soil/groundwater contamination^9, 14–16^. Water is unfit for drinking or other uses when it is contaminated or polluted due to pollutants such as industrial waste, agriculture, clinics, organic/inorganic chemicals and domestic sources^2, 17–19^.

Dyes possess complex aromatic molecular structures, enabling them to attain more stability and, as such, causing them to be challenging to break down (biodegrade). Dyes are hazardous water contaminant since they can be detected in polluted wastewater from numerous sectors and creates harmful and toxic effects on receiving water, even in trace concentrations^20^. Each year, more than 50 billion tonnes of dyes are anticipated to be used in the dyeing process, with reactive dyes accounting for over 30% of all dyes consumed globally. However, 20% and 60% of reactive dyes are lost throughout the dyeing process^21, 22^. Anionic dyes such as methyl orange (MO)^23–28^ and eosin yellow (EY)^29–33^ are one of them. Dyes are divided into three types—anionic, cationic, and non-ionic (dispersed dyes). In the wool and silk industries, eosin yellow (EY) dyes provide a red colour with a yellow fluorescence^34^. Methyl orange (MO) is utilised in printing, food, textile and pharmaceutical sectors and scientific research. Due to its −N=N− structure and low biodegradability, it may result in numerous human health and environmental problems^35, 36^. Dyes negatively affect human life, play a significant role in industrial wastewater and have a low degradation rate^37^. According to methyl orange 2022 market analysis, methyl orange is projected to grow at a CAGR of 5.5% from 2022 to 2030. The market growth is attributed to the increasing demand for methyl orange in laboratories, industries and agricultural applications. Also, the global Eosin market size was valued at USD Million in 2022 and will reach USD Million in 2028, with a CAGR from 2022 to 2028*.* Consequently, there is an urgent need to address the dye industry's challenge of removing toxins from water bodies and the environment.

The discovery and fabrication of new adsorbents for purifying hazardous pollutants from consumption water is essential for accomplishing the United Nations Sustainable Development Goals 6 (clean water and sanitation)^38^. Fabricating rapid techniques for synthesising low-cost, effective dye removal adsorbents is essential in wastewater purification^9^. Researchers investigated various methods for removing colour dye from wastewater^39^. The methods are ozonation^40, 41^, nanofiltration^42^, calcined alunite^43^; cloud point extraction^34, 44^, chemical coagulation/flocculation and oxidation processes^45, 46^; and adsorption^39, 47^. Adsorption is an excellent and significant method for treating industrial waste effluents because of its low cost, availability, economic viability, ease of operation and effectiveness^48, 49^. Zeolite is a porous hydrated mineral alumina silicate with chemical and physical cation exchange, molecular sieving and catalysis, and has high adsorption capacity^50^. It is employed as an adsorbent because of its large surface area, low production cost and raw material availability^51^. Zeolite possesses high ion exchange capacity, greater selectivity and specificity, and better radiation resistance. It has also shown advantages in immobilization and final disposal compared with organic ion exchangers^52^. This study uses eosin (EY) and methyl orange (MO) dye as adsorbates. These dyes were chosen based on their use in food, textile, pharmaceutical, paper printing and research facilities. Mono-azo groups in MO and EY and their low biodegradability raise environmental and public health concerns^53^. Due to its toxicity, removing these dyes from the water body is urgently needed. Adsorbents of various types are employed for dye removal. Although activated carbon is the most widely used adsorbent for removing a wide range of dyes, it is extremely expensive and difficult to regenerate^54^. Therefore, there is a need to develop alternative low-cost adsorbents in order to make the adsorption process a feasible wastewater treatment method.

To the authors' awareness, synthesised zeolite Y has not been thoroughly researched for removing MO and EY dye, except rebuffed. The main objective of this current study is to investigate and test the feasibility of removing MO and EY dyes from an aqueous solution using synthesised zeolite Y. The effect of various operating parameters on the adsorption of MO and EY dye in the batch process was investigated, including adsorption time, temperature, adsorbent dose, adsorbate concentration and pH. Furthermore, adsorption isotherms, kinetic models and thermodynamics were also determined.

## Material and experimental procedure

### Materials

Kaolin was procured from Arobieye village in Ado-Odo, Ota, Ogun state, Nigeria. With Sodium hydroxide pellets (Sigma-Aldrich, Lobal Chemie, ≥ 98%), concentrated sulfuric acid (Sigma-Aldrich, Lobal Chemie, 98%), Methyl orange (MO) (Sigma-Aldrich, ACS reagent, 99%), Eosin yellow (EY) (Merck chemical, > 99% purity). All these chemicals were of analytical grade and were used as received without further purification. Distilled water was used for the preparation of the solution.

### Synthesis of zeolite Y

Kaolin was purified using the wet beneficiation method to remove the impurities and then air dried for 2 days. The purified kaolin was calcined at 850 °C for 6 h to convert kaolin to metakaolin, as reported by Babalola et al.^55^. This was because Si–O or Al–O tetrahedral and octahedral structures possessed by kaolin are inactive to activation or modification, which can prevent the direct production of zeolites. As a result, kaolin must undergo thermal transformation to convert the inert phase to the active phase at higher temperatures by adding an alkali hydroxide^56–58^. The metakaolin was dealuminated by using concentrated sulphuric acid to reduce the composition of alumina and have the desired silica-to-alumina molar ratio required for the synthesis of zeolite Y. Dealuminated metakaolin was washed several times with distilled water to remove unreacted chemical and adjust the pH to 7. Sodium hydroxide pellets were then reacted with dealuminated kaolin at a ratio of 2.5:1 by weight and molar composition of 6SiO_2_:Al_2_O_3_:9Na_2_O:24H_2_O^50^. The gel obtained was aged 7 days at room temperature and then hydrothermally crystallised at 100 °C for 24 h.

### Batch equilibrium adsorption

Analytical chemical reagents were used without further purification in this study. MO and EY dyes were the adsorbates used. The MO and EY, dye solution concentrations were determined by a spectrophotometer (Shimadzu UV-160A) at 463 and 517 nm absorbance wavelengths, respectively. 1 g of MO and EY were dissolved separately in 1 L of distilled water to prepare a stock solution, and a serial solution of 20–60 mg/L was then used in the adsorption batch process. Synthesised zeolite Y was synthesised from kaolin deposits at Ogun state, Nigeria. Kaolin was subjected to several processes to synthesise zeolite Y with a Silica/Alumina molar ratio of 3.46; the procedure was described by^50^. The MO and EY dye concentrations were calculated at 463 and 517 nm absorbance wavelengths, respectively. Equation (1) was used to calculate the quantity of eosin yellow and methylene blue adsorbed at equilibrium, qe (mg/g):

1 $${q}_{e}=V\left(\frac{{c}_{0}-{c}_{e}}{m}\right)$$

The quantity of eosin yellow and methyl orange adsorbed (q_t_) at a time interval (t) was estimated using Eq. (2):

2 $${q}_{t}=V\left(\frac{{c}_{0}-{c}_{t}}{m}\right)$$

where m = weight of adsorbent (g), V = volume of adsorbate (L), C_o_ and C_e_ are the initial concentration of adsorbate and equilibrium concentration of adsorbate (mg/L). C_t_ = concentration of the adsorbate any time t (mg/L).

### Characterisation of Zeolite Y

The Nexus 470, Thermo Nicolet FTIR spectra USA model was used to determine the type of bonds in the sample over the 4000–400 cm^−1^ range. Images of zeolite Y were captured using scanning electron microscopy (SEM)-EDS model JOEL-JSF7600F.

### Experimental adsorption studies

Adsorptive removal of MO and EY dye from aqueous solutions by synthesised zeolite Y adsorbent was investigated to determine the influence of adsorption time, temperature, adsorbent dose and pH. The influence of adsorption time on the adsorption system was studied by adding 100 mL solution containing (20, 30, 40, 50 and 60 mg/L) of MO and EY to each 0.1 g zeolite Y sample. At various temperatures (20, 30, 40, 50 and 60 °C), the samples were shaken using a shaker (Rotaterm orbital and linear chakra). Adsorption time ranging from 2 to 90 min, and the filtrate was separated from the spent zeolite Y. The influence of pH on MO and EY was investigated by combining 0.1 g of zeolite Y with 100 mL of MO and EY dye (30 mg/L) solution separately. The pH was adjusted using a solution of 0.1 M HCl and 0.1 M NaOH, and the mixture was then stirred for 1 h at 20 °C. The influence of adsorbent dosage on removing MO and EY dye at C_o_ = 30 mg/L was also investigated with various adsorbent weights (0.1–0.5 g). The initial methyl orange and Eosin yellow concentrations (20–60 mg/L) were varied at 293 K, while adsorbent weight was held at 0.1 g/L for the adsorption isotherm investigations. The kinetic studies were carried out at 20 °C with an initial methyl orange and eosin yellow concentration of (20, 30, 40, 50, and 60 mg/L). Samples were collected at various shaking intervals until the methyl orange and eosin yellow concentrations reached equilibrium. Finally, the effect of temperature was investigated with 100 mL dye solution, 0.1 g adsorbent dosage and an adsorption time of 1 h at various temperatures (20, 30, 40, 50, and 60 °C).

### Adsorption isotherms

The capacity of an adsorbent is determined by its ability to remove the contaminant. The adsorption capacity is determined by mass per mass basis; the weight of adsorbed contaminant per adsorbent weight is the adsorption strength of the adsorbent. Although unit analysis can produce a unitless quantity, the adsorption capacity, q, is commonly expressed in mg/g units. Variations in adsorbent, pH, contaminant concentration, and temperature will affect a specific contaminant's equilibrium adsorption capacity. Variation in adsorbent quantity, equilibrium adsorption capacity remains constant. Adsorption equilibrium studies offer information on the capacity of the adsorbent. Adsorption isotherms express the adsorbent's surface properties and affinity and are defined by constant values. Isotherms can determine the comparison of adsorptive capacities of adsorbents for various pollutants. Adsorption isotherms, which serve as the foundation for designing adsorption systems, can be used to analyse equilibrium data^59–61^.

#### Langmuir isotherm

Irving Langmuir isotherm focused on gases adsorbed on the solid surface, and it was derived from a proposed kinetic mechanism of Langmuir^62^.

The Langmuir postulates are as follows:

1. The adsorbent's surface features uniformly energetic adsorption sites.
2. The molecules striking the adsorbent surface and adsorbed
3. Adsorbed molecules did not interact with each other.
4. The adsorption extent is less than one complete mono-molecular layer on the surface i.e., monolayer coverage.
5. Each adsorbed complex has the same structure because they have the same mechanism.

The Langmuir isotherm is given by^59^:

3 $$q_{e} = \frac{{Q_{0} bC_{e} }}{{1 + bC_{e} }}$$

Equation (3) is then linearised as shown below:

4 $$\frac{{C_{e} }}{{q_{e} }} = \frac{1}{{Q_{0} }}C_{e} + \frac{1}{{bQ_{0} }}$$

where b and $Q_{0}$ represent Langmuir constant (L/mg) and adsorption maximum capacity (mg/g). ${C}_{e}$ and *qe* denote the amount of aqueous solution at equilibrium (mg/L) and the amount of aqueous solution absorbed by an adsorbent at equilibrium (mg/g). A plot of $Ce/{q}_{e}$ versus ${c}_{e}$ was used to calculate $Q_{0}$, and b.

#### Freundlich isotherm model

Adsorption on heterogeneous surfaces in dilute liquids is simulated using the Freundlich empirically derived model^63^. Freundlich was the first to develop a mathematical model for adsorption onto solid surfaces, and his equation is still one of the most frequently cited adsorption isotherms today. The isotherm is given by^64^.

5 $${ q}_{e}= {K}_{f }{c}_{e}^\frac{1}{n}$$

where ${K}_{f}$ and *n* is the Freundlich adsorption constant and adsorption intensity (an empirical constant). The isotherm can be linearised to linear forms as shown below:

6 $$\mathrm{ln}{q}_{e}=\mathrm{ln}{K}_{f}+\frac{1}{n}\mathrm{ln}{Cc}_{e}$$

The adsorption constant was determined by a plotted graph of $\mathrm{ln}{q}_{e}$ versus $\mathrm{ln}{c}_{e}$, which gives the value of ${K}_{f}$ and *n.* A straight-line graph was obtained with a slope of 1/*n* and the intercept on $\mathrm{ln}{q}_{e}$ axis equal to $\mathrm{ln}{K}_{f}$.

## Results and discussion

### Zeolite Y characterization

**Figure 1:**
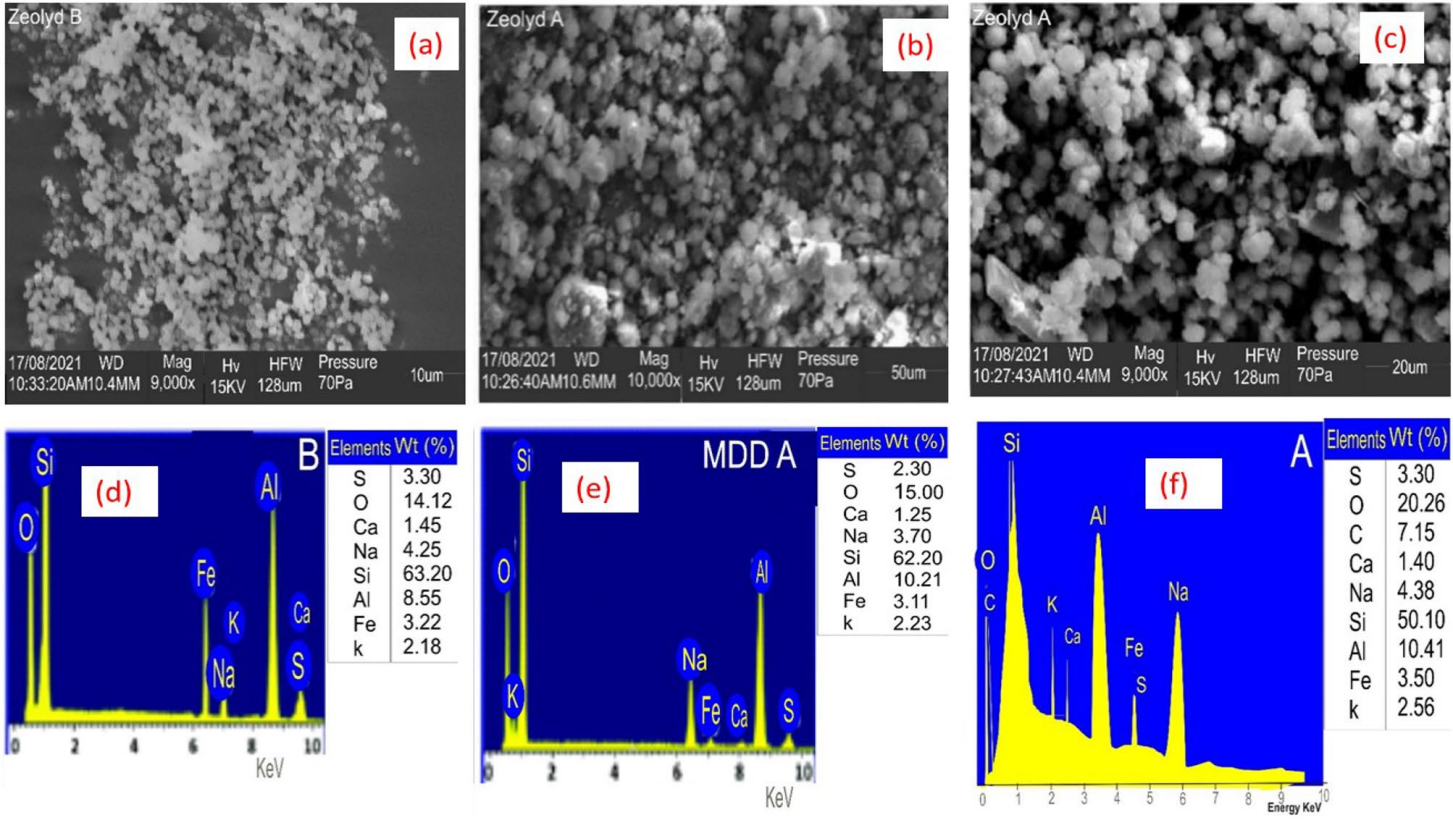
(**a**) SEM before adsorption, (**b**) SEM after adsorption of methyl orange, (**c**) SEM after adsorption of eosin yellow, (**d**) EDX before adsorption, (**e**) EDX after adsorption of methyl orange, (**f**) EDX after adsorption of eosin yellow.

**Figure 2:**
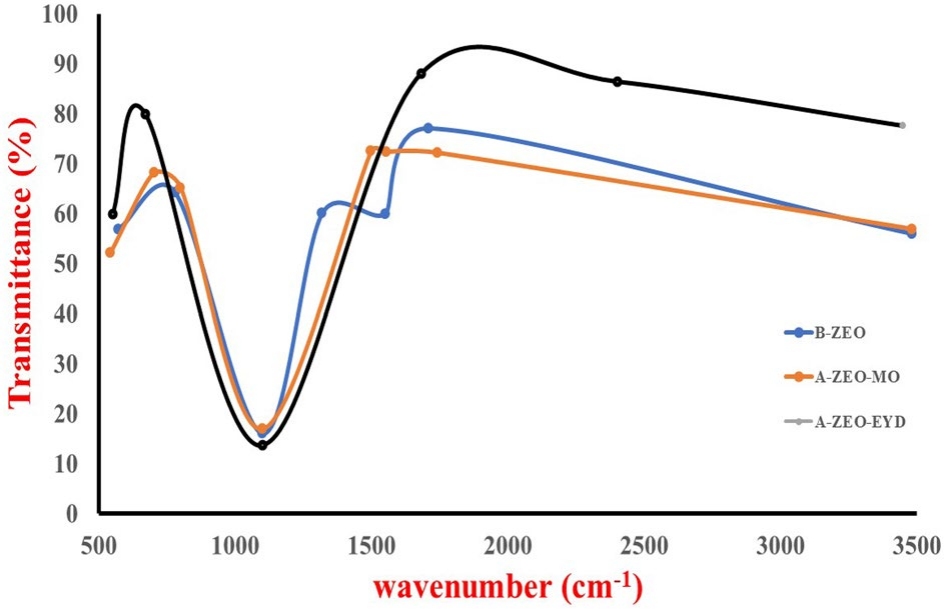
Spectra FTIR features of zeolite Y before and after adsorption with methyl orange and eosin yellow dye.

**Figure 3:**
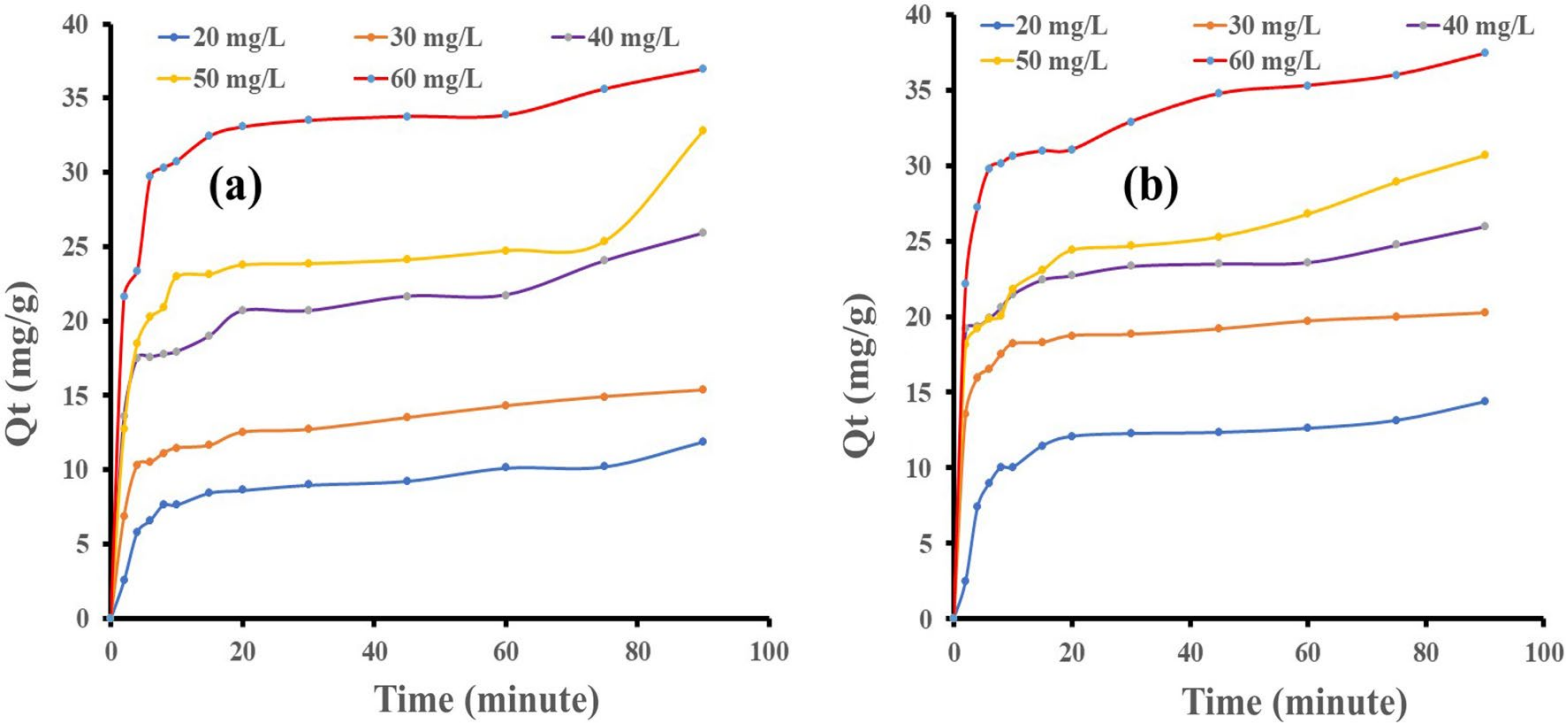
(**a**) Effect of contact time on the amount adsorbed of eosin yellow, (**b**) effect of contact time on the amount adsorbed of methyl orange at (temperature = 303 K, agitation speed = 100 rpm, volume = 100 mL, weight = 0.1 g).

The SEM microphotographs of the zeolite Y samples are shown in Fig. 1a–c. Figure 1a shows the SEM before adsorption, and Fig. 1b,c highlights the SEM image after methyl orange and eosin yellow dye adsorption. The image has pores of various sizes that allow the dyes methyl orange and eosin yellow to adhere to its surface, as shown in Fig. 1a. Figure 1a shows the bright spots and the adsorbent's rough, porous surface, which enhances adsorption capacity. Figure 1b,c shows that the dye covered the adsorbent's pores, caves and surfaces. Figure 1d–f shows the EDX analysis before and after methyl orange and eosin yellow adsorption unto zeolite Y. As seen in Fig. 1d–f, EDX results show that the main components of zeolite Y are Si, Al, O, and Na^65–67^. The Aluminium in the zeolite Y in Fig. 1d shifted from 8.55 to 10.21 and 10.41 in Fig. 1e,f, meaning the silicon quantity has reduced. The surface area was estimated using Brunauer–Emmett–Teller (BET). Accordingly, the surface area, pore size, and pore volume of Zeolite Y before adsorption (445.36 m^2^/g, 30.53 Å, 0.60 cm^3^/g) and after adsorption for methyl orange (438.25 m^2^/g, 23.79 Å, 0.60 cm^3^/g) and eosin yellow (442.67 m^2^/g, 25.53 Å, 0.60 cm^3^/g), respectively. The surface area of the adsorbent was reduced after adsorption because the dyes were adsorbed on the surface of the adsorbent. Table 1 depicts the BET results before and after loading. The FT-IR spectra of the zeolite Y adsorbent before and after loading are shown in Fig. 2. The bands at 3480.72, 1710.18, 1550.31, 1318.25, 780.13 cm^−1^ corresponding to O–H, C–H, –CH_3_, –COOH, –OH are present in zeolite Y. The peak at 3480.72 cm^−1^ is stretching vibrations of a hydroxyl group (O–H), 1710.18 cm^−1^ is C–H stretching vibration, and 1550.31 cm^−1^ is CH_3_ asymmetric deformation of the connection of the CH_2_ group^68^. The bands at 1318.25 and 780.13 cm^−1^ are COOH asymmetric stretching vibrations in the carboxyl group and OH` bending vibration of the carboxylic functional group. The band around 1100.28 cm^−1^ is likely attributed to the presence of asymmetrical stretch vibration of Si–O–Si and Si–O–Al stretching vibrations and at 571.46 cm^−1^ attributed to the presence of Si–O and Al–O bending vibration modes, as shown in Fig. 3 for zeolite Y before adsorption (B-ZEO). There is a significant difference in the FTIR before and after the adsorption of MO (A-ZEO-MO) and eosin yellow dye (A-ZEO-EYD), as shown in Fig. 3. The band at 1710.18 cm^−1^ (B-ZEO) shifted to 1742.30 cm^−1^ (A-ZEO-MO) and is attributed to C–H stretching vibration. The band at 780.13 cm^−1^ shifted to 800.15 cm^−1^ due to the carboxylic functional group's OH– bending vibration. Additionally, the band at 3480.72 cm^−1^ (B-ZEO) shifted to 3448.50 cm^−1^ (A-ZEO-EYD) due to O–H stretching vibrations of Silicon (Si–OH) and hydrogen bonding with other silicon or water molecules. The band at 571.46 cm^−1^ (B-ZEO) shifted to 550.15 cm^−1^ (A-ZEO-EYD) due to Si–O–Si symmetric stretching vibrations of bridge bonds and O–Si–O bending vibrations. Within the spectrum of the zeolite Y after adsorption, the band at 1318.28 cm^−1^ (B-ZEO) shifted to 1498.55 cm^−1^ (A-ZEO-MO), suggesting that the carboxylic acid functional group on MO was linked to an amino group in the zeolite Y^69, 70^.

**Table 1:** BET results before and after adsorption.

Sample	Surface area (m^2^/g)	Pore size (Å)	Pore volume (cm^3^/g)	Average pore diameter (nm)
Zeolite Y	445.36	30.53	0.60	30.53
Zeolite Y with eosin	442.67	19.34	0.60	30.53
Zeolite Y with MO	442.61	25.53	0.60	30.53

### Effects of operational parameters on methyl orange and eosin yellow dye Adsorption

#### Contact time

Figure 3a,b demonstrate the effect of contact time on the quantity of dyes adsorbed. It was observed that the amount of MO and EY uptake is increased with increasing contact time at all initial dye concentrations. Furthermore, the amount of dye adsorbed increases with the initial dye concentration. The adsorption uptake for the first 30 min was rapid, then proceeded slower. As time proceeds, the dye concentration is reduced due to the accumulation of dye particles in the vacant sites, leading to decreased adsorption. At 60 min, the percentage removal of MO and EY onto zeolite Y was 63%, 61%, 59%, 53, and 59% for MO and 51%, 48%, 54.30%, 49.50%, and 56.40% for EY at concentrations of 20, 30, 40, 50, and 60 mg/L, respectively. At 90 min, the percentage removal of MO and EY onto zeolite Y was 80%, 67%, 65%, 61%, and 62% for MO and 60%, 51.20%, 64.70%, 65.60%, and 61% for EY at concentrations of 20, 30, 40, 50, and 60 mg/L, respectively. The large percentage removal at low concentrations is due to the availability of more active adsorption sites on the zeolite with less MO and EY molecules to occupy, so the limited available MO and EY molecules were adsorbed rapidly^71, 72^.

#### pH value

**Figure 4:**
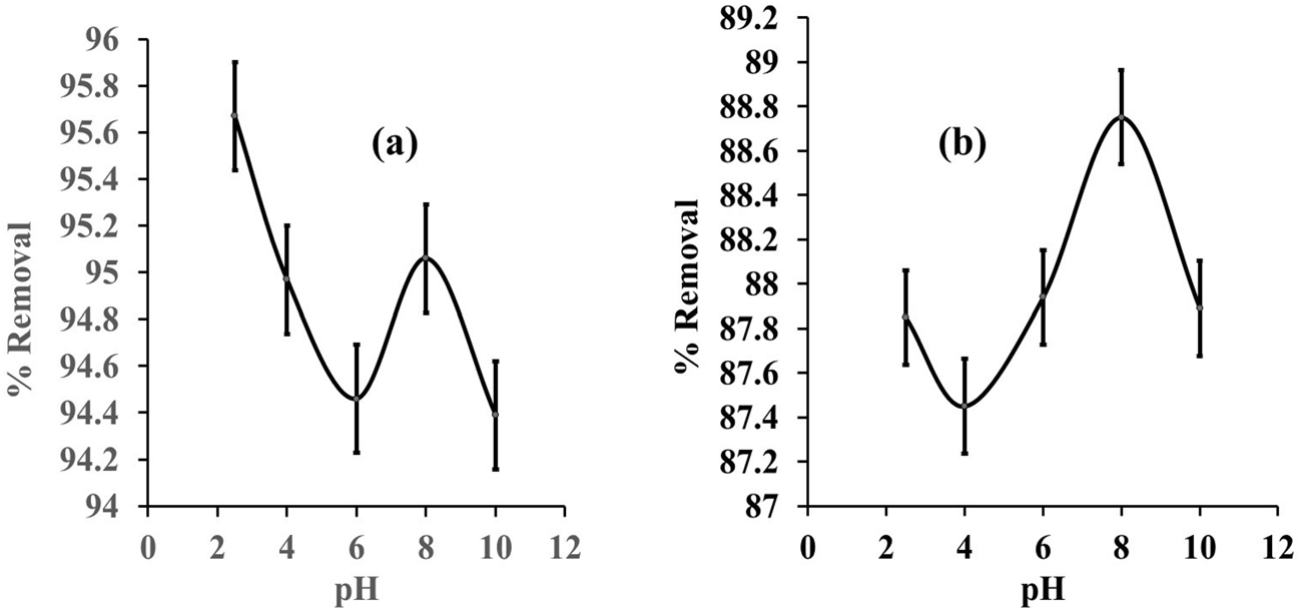
Influence of pH on the removal of (**a**) eosin yellow dye, (**b**) methyl orange on zeolite Y (adsorbent dose = 0.1 g, initial concentration of both adsorbates = 50 mg/L, contact time = 60 min, agitation speed = 150 rpm, temperature = 303 K).

Figure 4a,b show the effect of pH on removing eosin yellow and methyl orange dyes from aqueous solutions at various pH levels. When the pH was varied from 2.5 to 10.0, eosin yellow and methyl orange had the greatest adsorption capability at pH 2.5 and 8. The maximum adsorption was observed at pH 2.5 with 95.70% for eosin yellow and pH 8 with 87.90% for methyl orange. These findings could be explained by the differences in surface charge and dye ionisation between the dyes (eosin yellow and methyl orange) and zeolite Y. The lower pH increases H^+^ ion concentration in an acidic medium, and the zeolite Y surface becomes more positively charged.

In contrast, in an alkaline medium, the higher pH OH^−^ ion concentration and the zeolite Y surface become more negatively charged. The strong electrostatic attraction between the anionic methyl orange molecule and the positively charged adsorption site results in the high adsorption of methyl orange dye. Also, the strong electrostatic attraction between the cationic eosin yellow molecule and the negatively charged adsorption site results in high adsorption of eosin yellow dye^73^.

#### Adsorbent dosage

**Figure 5:**
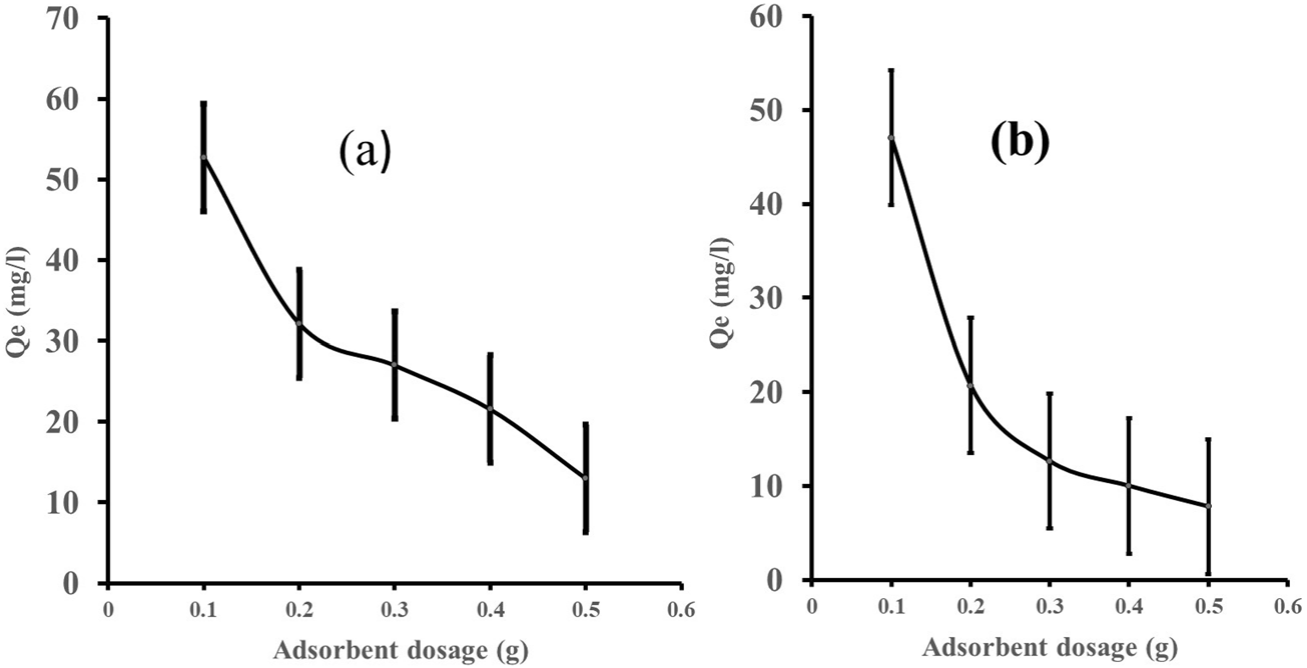
(**a**) Adsorbent dosage on eosin yellow dye adsorption by zeolite Y, (**b**) adsorbent dosage on methyl orange dye adsorption by Zeolite Y (C_o_ = 30 mg/L, temperature = 303 K, time = 60 min, V = 100 mL).

The adsorbent dose is also a significant criterion in adsorption studies because it involves effective adsorbate removal while saving money. Figure 5 describes the effect of adsorbent weight (zeolite Y), M, on equilibrium adsorption capacity (Qe). Figure 5a,b shows that as the adsorbent dosage is increased, the adsorption capacity decreases. This is due to the active sites being exposed to a small quantity of adsorbent while a few fractions were exposed to a higher dose of the zeolite Y^72, 74^. This will influence the increase in the percentage removal of the dyes (methyl orange and eosin yellow). An increase in adsorbent mass leads to increased active sites^75^.

#### Temperature effect

**Figure 6:**
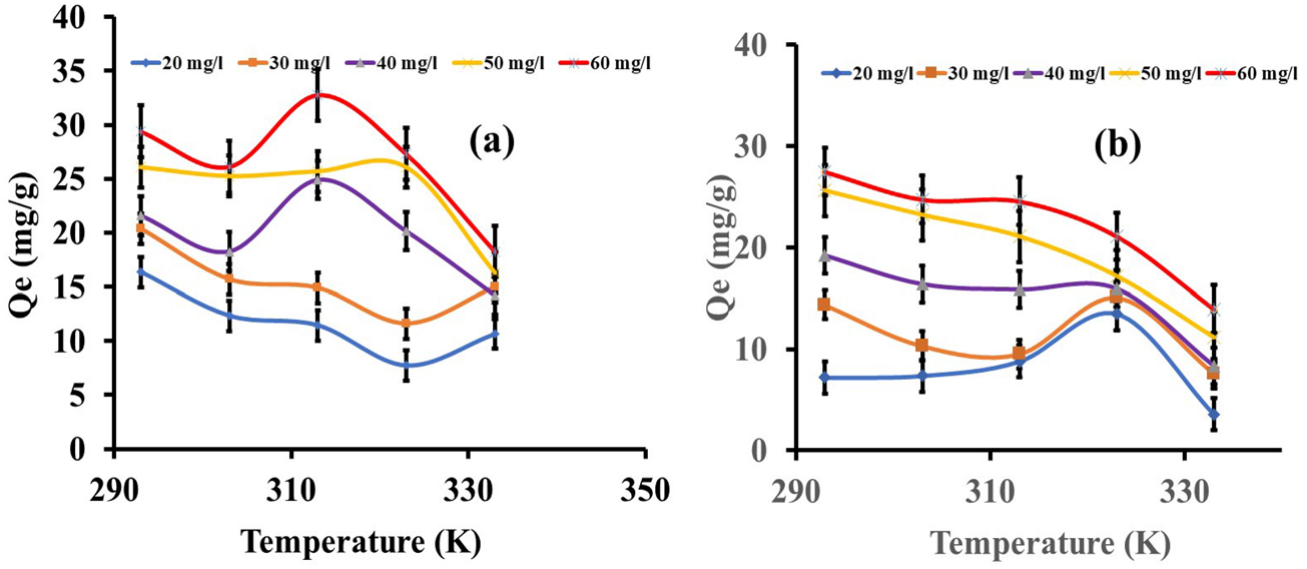
Effect of temperature on the (**a**) eosin yellow, (**b**) methyl orange on zeolite Y (adsorbent dose = 0.1 g, initial concentration of adsorbate = 100 mg/L, contact time = 60 min, agitation speed = 100 rpm).

The influence of temperature on eosin yellow and methyl orange adsorption using zeolite Y as an adsorbent was studied. The adsorption capacity for the dyes is illustrated in Fig. 6a,b. The zeolite Y's adsorption capacity decreases as the temperature rises from 293 to 333 K. The amount adsorbed decreases from 16.35 to 10.66 mg/g at 20 mg/L, 20.39 to 14.99 mg/g at 30 mg/L, 21.57 to 14.19 mg/g at 40 mg/L, 26.08 to 16.32 mg/g at 50 mg/L and 29.39 to 18.27 mg/g at 60 mg/L for eosin yellow. Likewise for methyl orange, the amount adsorbed decreases from 7.20 to 3.58 mg/g at 20 mg/L, 14.37 to 7.56 mg/g at 30 mg/L, 19.24 to 8.35 mg/g at 40 mg/L, 26.61 to 11.19 mg/g at 50 mg/L, and 26.61 to 11.19 mg/g at 60 mg/L, respectively. These obvious trends support the notion that adsorption is advantageous at low and detrimental at high temperatures. This also implied an exothermic reaction corresponding to the estimated thermodynamic parameters^71, 72^. The amount of EY and MO adsorbed reduced as the temperature increased, which has a negative effect on eosin yellow and methyl orange adsorption on zeolite Y, demonstrating an inverse link between temperature and percentage removal and adsorption capacity of the adsorption system^76^.

### Adsorption isotherm studies

**Figure 7:**
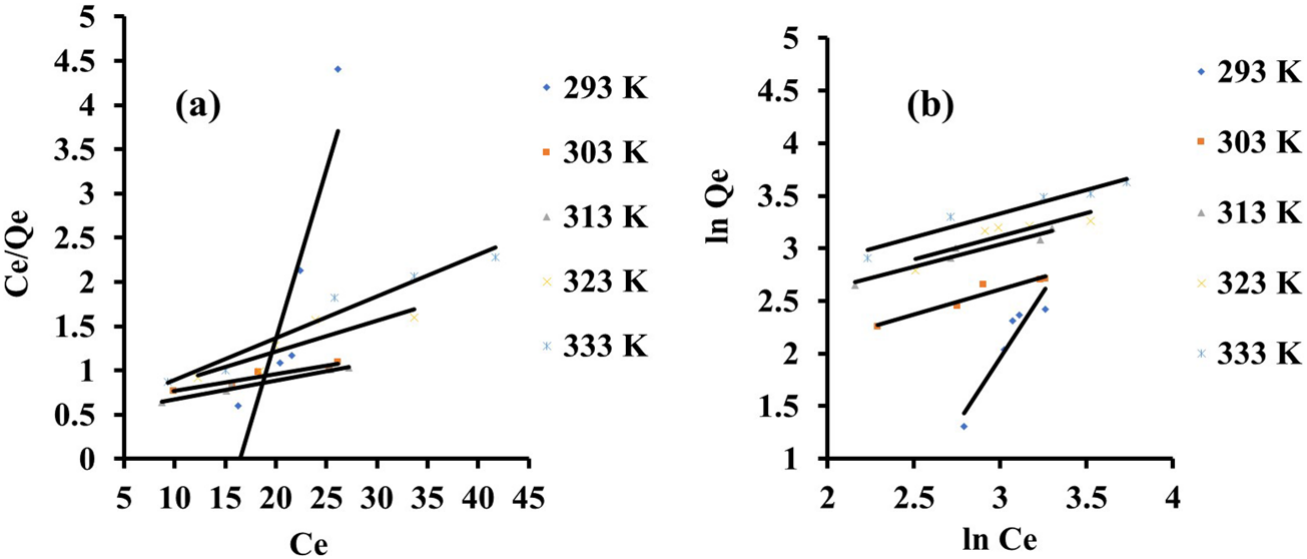
Graphs for (**a**) Langmuir (**b**) Freundlich isotherm for the adsorption of eosin yellow (agitation speed = 140 rpm; pH 2.5; reaction time = 60 min; adsorbent weight = 0.1 g, tempt = 293 K).

**Figure 8:**
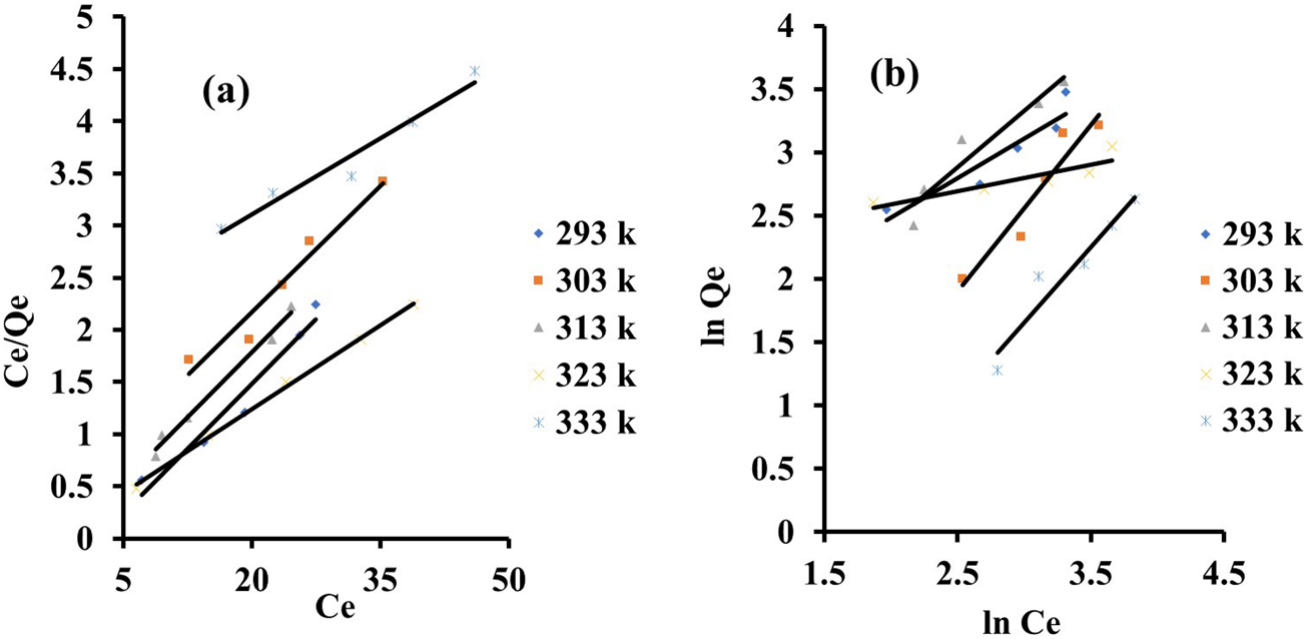
Graphs for (**a**) Langmuir (**b**) Freundlich isotherm for the adsorption of methyl orange (agitation speed = 140 rpm; pH 8; reaction time = 60 min; adsorbent = 0.1 g).

Adsorption process design and optimisation require the development of an appropriate isotherm model. The adsorption isotherms parameter of eosin yellow and methyl orange dye onto zeolite Y were studied with the following model as shown in Figs. 7a,b and 8a,b. The results of the isotherm are presented in Tables 2 and 3. The adsorption system is better fitted in the Langmuir isotherm for methyl orange and best fitted with Freundlich isotherm for eosin yellow compared to the coefficient regression (R^2^). This implies that the adsorption system is monolayer and homogeneous for methyl orange and multilayer and heterogenous for eosin yellow, with maximum adsorption capacities for eosin yellow and methyl orange being 52.91 and 20.62 mg/g, respectively. Due to its ionic properties, eosin yellow dye has a higher uptake capacity than methyl orange dye; eosin yellow has a higher adsorption capacity, indicating that it is more attainable towards the porous adsorbent structure, as suggested by its lowest hydrated radii value. The R_L_ value is within the 0 < RL < 1 range, implying that the adsorption process is favourable. In addition, the isotherm model was further justified with lower values of the sum of absolute error (EABS), the sum of square error (SSE), and chi-square (χ^2^) obtained, which also obeyed Langmuir isotherm for methyl orange and Freundlich isotherm for eosin yellow.

**Table 2:** Isothermal constants of different isotherms for the adsorption system of eosin yellow dye onto zeolite Y.

Isotherms	Parameters	293 K	303 K	313 K	323 K	333 K
Langmuir	Q_o_ (mg/g)	2.60	52.91	47.17	28.49	21.23
	B (L/mg)	6.38	30.75	22.02	14.61	9.02
	R^2^	0.81	0.91	0.98	0.77	0.96
	R_L_	0.008	0.002	0.002	0.003	0.006
	x^2^	0.42	34.59	31.33	9.31	6.65
	SSE (%)	0.29	11.86	10.66	2.58	1.85
	EABS	1.05	42.77	38.44	16.22	11.89
Freundlich	K_F_	0.004	3.28	5.76	5.91	7.31
	1/n	2.53	0.47	0.43	0.45	0.45
	R^2^	0.88	0.92	0.94	0.75	0.94
	x^2^	3697.50	14.32	1.53	6.85	0.56
	SSE (%)	1.01	1.90	0.82	1.76	0.16
	EABS	3.65	6.86	2.97	6.36	2.03

**Table 3:** Isothermal constants of different isotherms for the adsorption system of methyl orange dye onto zeolite Y.

Isotherms	Parameters	293 K	303 K	313 K	323 K	333 K
Langmuir	Q_o_ (mg/g)	12.06	12.36	11.96	18.59	20.62
	B (L/mg)	-0.46	0.15	0.50	0.33	0.02
	R^2^	0.95	0.95	0.99	1.00	0.96
	R_L_	0.12	0.26	0.09	0.13	0.69
	x^2^	0.05	0.002	0.05	7.85	0.86
	SSE (%)	0.20	0.07	0.21	3.35	1.16
	EABS	0.74	0.26	0.76	12.08	4.20
Freundlich	K_F_	3.41	0.25	1.93	8.79	0.15
	1/n	0.63	1.32	0.89	0.21	1.19
	R^2^	0.87	0.91	0.92	0.80	0.93
	x^2^	4.22	615.65	44.40	0.59	1797.70
	SSE (%)	1.05	3.43	2.57	0.63	4.51
	EABS	3.79	12.37	9.27	2.28	16.27

### Adsorption kinetic studies

**Figure 9:**
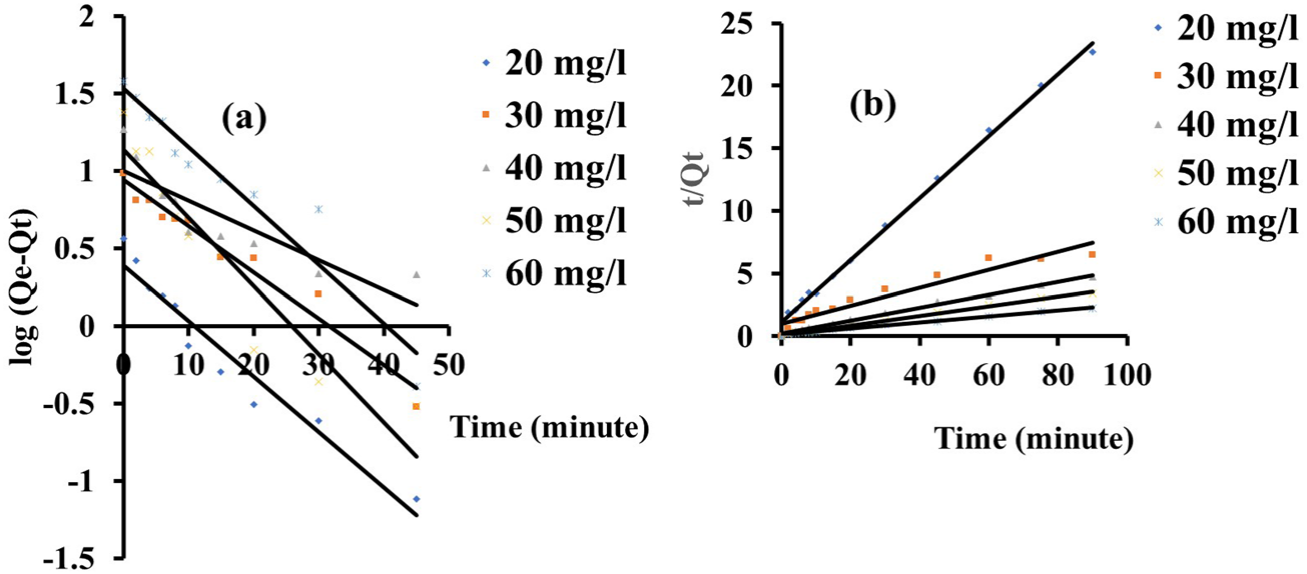
Graphs for (**a**) Pseudo-first order (**b**) Pseudo-second order for adsorption of eosin yellow (pH 2.5; agitation speed = 100 rpm; adsorbent dose = 0.1 g, temperature = 293 K).

**Figure 10:**
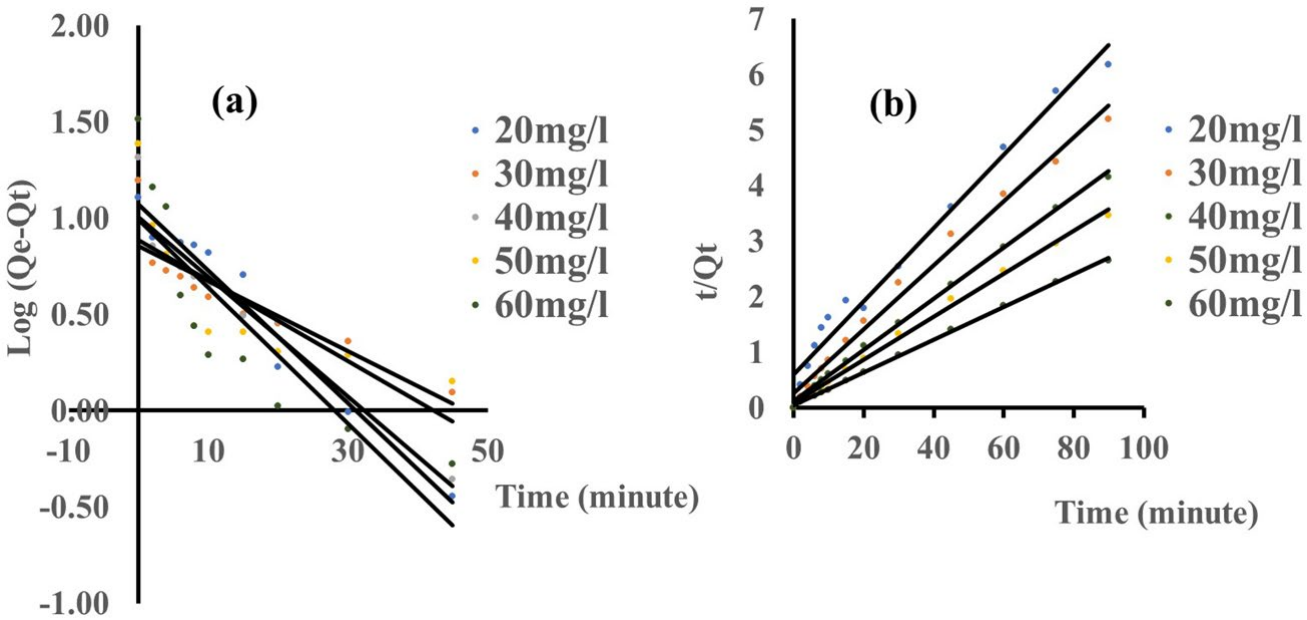
Plots for (**a**) Pseudo-first order and (**b**) Pseudo-second order for adsorption of methyl orange (pH 8; agitation speed = 100 rpm; adsorbent dose = 0.1 g, temperature = 293 K).

The adsorption kinetic investigation for the eosin yellow system was carried out by altering the adsorption time between 30 and 90 min. The adsorption kinetic model parameters of eosin yellow and methyl orange dye onto zeolite Y were studied with the following model as shown in Figs. 9a,b and 10a,b. The first-order, second-order pseudo and the error analysis results are presented in Tables 4 and 5. According to their regression coefficient (R^2^) and possible chemical interaction, results showed that the second-order pseudo model was best fitted when compared to the first-order pseudo models for both dyes, as the adsorption capacity of the experimental (${q}_{e,exp}$) was relatively close to the calculated (${q}_{e,cal}$) in second-order pseudo than first-order pseudo. In addition, the model was further justified with lower values of the sum of absolute error (EABS), the sum of square error (SSE), and chi-square (χ^2^) obtained, which also obeyed the second pseudo-second order.

**Table 4:** Show the kinetics model parameters for the adsorption of eosin yellow dye onto zeolite Y.

Kinetic model	Parameters					
	293 K					
	Co (mg/L)	20 mg/L	30 mg/L	40 mg/L	50 mg/L	60 mg/L
Pseudo-first order	q_oexpt_ (mg/g)	3.65	9.61	18.43	23.92	37.60
	R^2^	0.94	0.96	0.77	0.89	0.93
	q_ecal_ (mg/g)	2.46	8.70	9.99	13.68	34.11
	K_1_	0.08	0.07	0.04	0.10	0.09
	x^2^	0.58	0.10	7.13	7.67	0.36
	SSE (%)	0.33	0.25	2.34	2.84	0.97
	EABS	1.19	0.91	8.44	10.24	3.49
Pseudo-second order	R^2^	1.00	0.93	1.00	1.00	0.99
	q_ecal_(mg/g)	4.03	13.95	19.53	26.32	42.02
	q_oexp_(mg/g)	3.65	9.61	18.43	23.92	37.60
	K_2_	0.05	0.005	0.01	0.01	0.004
	X^2^	0.04	1.35	0.06	0.22	0.46
	SSE (%)	0.11	1.20	0.31	0.67	0.13
	EABS	0.38	4.34	1.10	2.40	4.42

**Table 5:** Show the kinetic model parameters for the adsorption of methyl orange dye onto zeolite Y.

Kinetic model	Parameters					
	293 K					
	Co (mg/L)	20 mg/L	30 mg/L	40 mg/L	50 mg/L	60 mg/L
Pseudo-first order	q_oexpt_ (mg/g)	12.80	15.63	20.76	24.39	32.53
	R^2^	0.96	0.80	0.93	0.61	0.74
	q_ecal_ (mg/g)	11.83	7.20	10.15	7.69	9.82
	K_1_	0.09	0.04	0.07	0.05	0.08
	x^2^n	0.08	9.87	11.09	36.33	52.55
	SSE (%)	0.27	2.34	2.94	4.63	6.30
	EABS	0.97	8.43	10.61	16.70	22.71
Pseudo-second order	R^2^	0.98	0.99	1.00	1.00	1.00
	q_ecal_ (mg/g)	15.17	17.39	21.79	25.84	33.90
	q_oexp_ (mg/g)	12.80	15.63	20.76	24.39	32.53
	K_2_	0.007	0.01	0.02	0.02	0.02
	x^2^	0.37	0.18	0.05	0.08	0.06
	SSE (%)	0.66	0.49	0.28	0.40	0.38
	EABS	2.37	1.76	1.03	1.45	1.37

### Thermodynamic studies

The change in the value of the thermodynamic equilibrium constant $({K}_{c})$, with temperature, can be used to estimate the enthalpy (${\Delta H}^{0}$), Gibb’s free energy (${\Delta G}^{o}$) and entropy (${\Delta S}^{o}$). The thermodynamic equilibrium constant,${K}_{c}$, was determined using the relation:

7 $${q}_{e}= {K}_{c}{C}_{e}$$

The change in Gibb’s free energy was thus calculated using:

8 $$\Delta G= -RTIn{K}_{c}$$

However,

9 $$\Delta G= \Delta H-T\Delta S-RTIn{K}_{c}$$

The temperature dependence of the Gibbs free energy change can be written as:

10 $$d\left(\frac{{\Delta G}^{\mathrm{o}}}{T}\right)= -\frac{{\Delta H}^{\mathrm{o}}}{{T}^{2}}dT$$

As a result, substituting Eq. (9) into Eq. (10) yields Eq. (11), equilibrium constant can be described as temperature-dependent adsorption enthalpy change.

11 $$d In {K}_{c}= -\frac{{\Delta H}^{\mathrm{o}}}{{RT}^{2}}dT$$

From Eq. (9).

12 $$In {K}_{c}=\frac{\Delta S}{R}-\frac{\Delta H}{RT}$$

**Figure 11:**
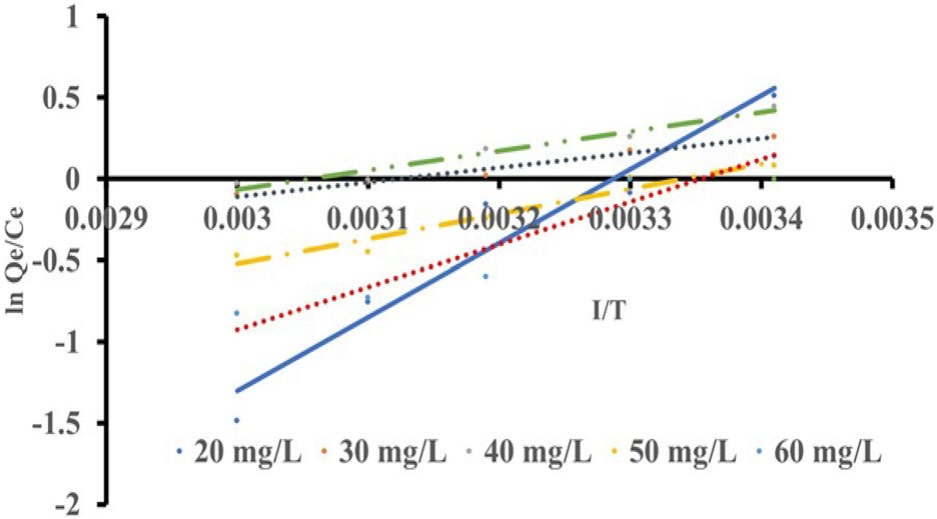
A plotted graph of $\mathrm{ln}\frac{{q}_{e}}{{C}_{e}}$ against $\frac{1}{T}$ for eosin yellow.

**Figure 12:**
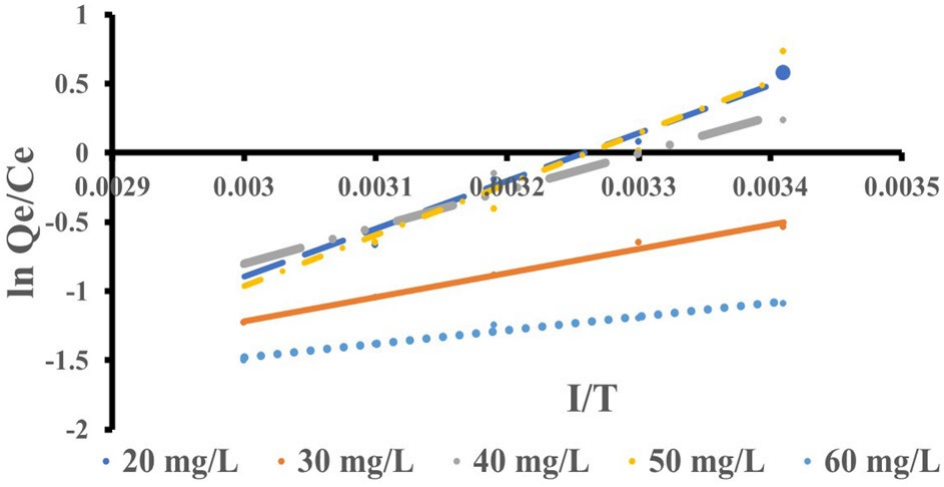
A plotted graph of $\mathrm{ln}\frac{{q}_{e}}{{C}_{e}}$ against $\frac{1}{T}$ for methyl orange.

Figure 11 and 12 show the plots of $\mathrm{ln}\frac{{q}_{e}}{{C}_{e}}$ Against ^1^/T for eosin and methyl orange, respectively. The slopes and intercept of the linear plot are used to determine the values of ${\Delta H}^{0}$ and ${\Delta S}^{o}$ (Table 6).

**Table 6:** Thermodynamic parameters for removing eosin yellow and methyl orange from aqueous solution by zeolite Y.

Parameter	Adsorbate	
	Eosin yellow	Methyl orange
${\Delta H}^{0}$ (KJ/mol)	− 11,049.22	− 8430.52
${\Delta S}^{o}$ (J/mol K)	− 123.95	− 93.74
${\Delta G}^{o}$ (KJ/mol) at 293 K	− 11,012	− 8403.06
R^2^	0.94	0.98

The ${\Delta H}^{0}$, ${\Delta S}^{o}$, and ${\Delta G}^{o}$ Values denote exothermic reaction, decrease in liquid–solid interfaces and spontaneous^77–79^. The negative values of ΔG° indicated the feasibility and spontaneity of the adsorption process without an induction period^80^.

### Adsorption mechanism

**Figure 13:**
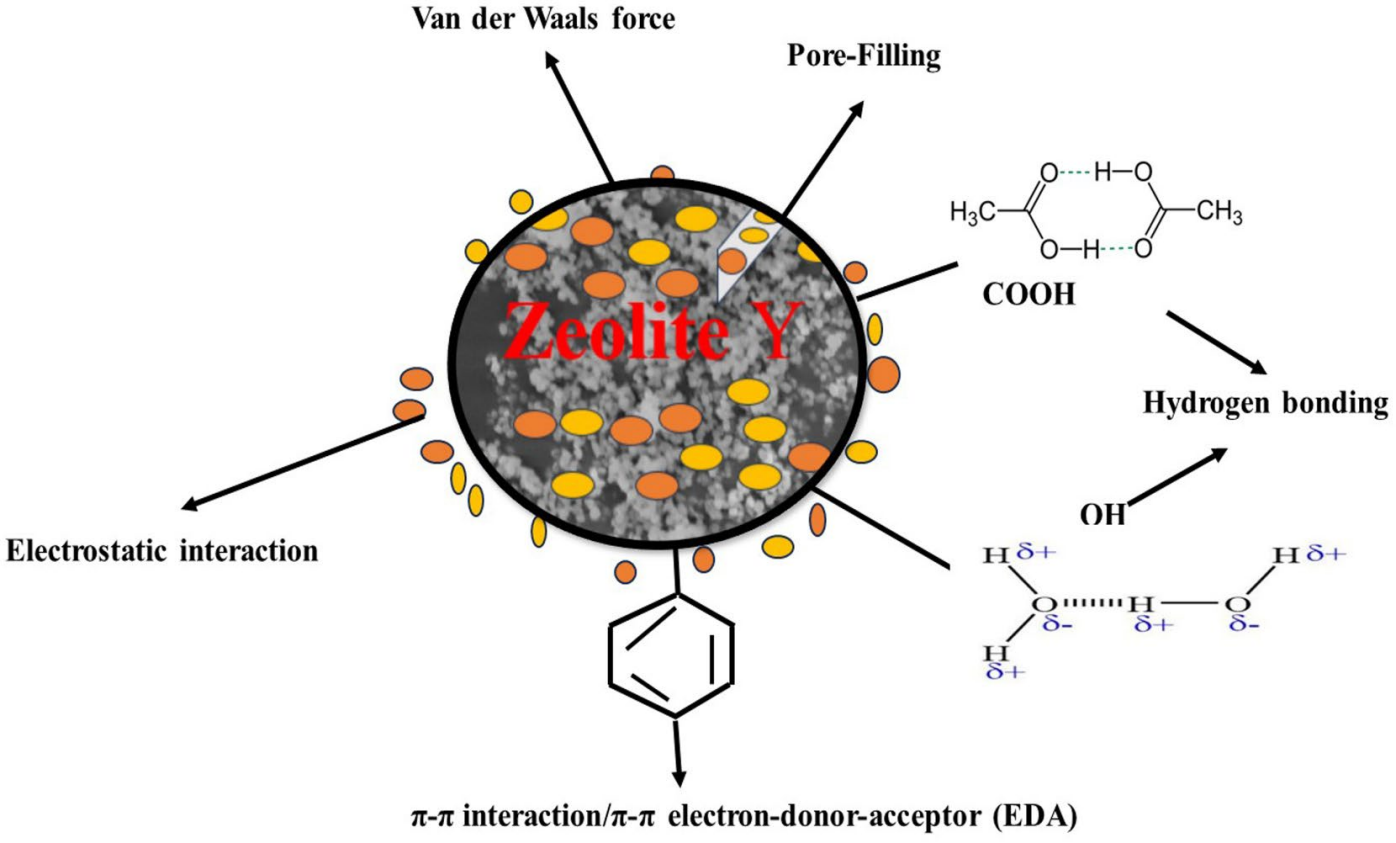
Adsorption mechanism of MO and EY onto zeolite Y.

The adsorption mechanisms of MO and EY are presented in Fig. 13. The mechanisms of adsorption are described in terms of electrostatic interaction, functional group, π–π electron–donor–acceptor (EDA)/π–π interaction, adsorbent textural, crystalline properties, structure, Van der Waals force, hydrogen bond interaction, and methyl orange and eosin yellow properties in aqueous solution. The interaction between the positively charged on the surface of zeolite Y and the negatively charged of the MO and EY increased the adsorption capability of zeolite Y to MO and EY^81, 82^. When proton acceptor and proton donor groups are engaged, carboxylic and hydroxyl groups on the surface of zeolite Y interact with MO and EY via hydrogen bonding^82–84^. MO and EY could possibly adsorb on the zeolite Y’s outer layer by π–π stacking^85^. The surface area and total pore size of the BET of zeolite Y before MB and EY dye adsorption were 445.36 m^2^/g and 30.5342 Å, respectively, while after adsorption they were 442.67 m^2^/g and 19.3421 Å for EY and 442.607 m^2^/g and 25.5342 Å for MO. The reduction in surface area and pore size following MB and EY dye adsorption suggests pore filling due to dye molecule occupation after adsorption.

## Conclusion

The current research involved using zeolite Y as an adsorbent to remove MO and EY dye from an aqueous solution. This research showed the potential of zeolite Y as an efficient adsorption performance. The well-developed zeolite Y porous structure, with BET surface area and total pore volume of 445.36 m^2^/g and 0.603567cm^3^/g, respectively, improves adsorption. Equilibrium studies showed that Langmuir isotherm best fits with methyl orange, while Freundlich isotherm was best described as the adsorption isotherm of eosin yellow. The pseudo-second-order kinetic model exhibited the best correlation for the experimental data. Thermodynamic studies show that adsorption was an exothermic reaction (enthalpy < 0) and feasible ($\mathrm{Gibbs free energy}< 0$) at the temperature under investigation. Finally, due to its high surface area, large adsorption capacity, and cost-effectiveness, zeolite Y prepared from kaolin seems to be an effective and efficient adsorbent for the removal of eosin yellow and methyl orange dye from aqueous solution as its raw material (kaolin) is readily available in different parts of the country (Nigeria). Further study should be done on the re-usability of zeolite Y and the continuous adsorption process.

## Data Availability

Data is available on request from the corresponding authors.
